# The Role of O-GlcNAcylation for Protection against Ischemia-Reperfusion Injury

**DOI:** 10.3390/ijms20020404

**Published:** 2019-01-18

**Authors:** Rebekka Vibjerg Jensen, Ioanna Andreadou, Derek J. Hausenloy, Hans Erik Bøtker

**Affiliations:** 1 Department of Cardiology, Aarhus University Hospital, Skejby, Palle Juul-Jensens Blvd. 99, 8200 Aarhus N, Denmark; heb@dadlnet.dk; 2 Laboratory of Pharmacology, Faculty of Pharmacy, National and Kapodistrian University of Athens Panepistimiopolis, 15771 Zografou, Greece; jandread@pharm.uoa.gr; 3 Cardiovascular & Metabolic Disorders Program, Duke-National University of Singapore Medical School, Singapore 169857, Singapore; derek.hausenloy@duke-nus.edu.sg; 4 National Heart Research Institute Singapore, National Heart Centre, Singapore 169609, Singapore; 5 Yong Loo Lin School of Medicine, National University Singapore, Singapore 119228, Singapore; 6 The Hatter Cardiovascular Institute, University College London, London WC1E 6HX, UK; 7 The National Institute of Health Research University College London Hospitals Biomedical Research Centre, Research & Development, London W1T 7DN, UK; 8 Tecnologico de Monterrey, Centro de Biotecnologia-FEMSA, Monterrey 64849, Mexico

**Keywords:** ischemia-reperfusion injury, O-GlcNAc, cardioprotection

## Abstract

Ischemia reperfusion injury (IR injury) associated with ischemic heart disease contributes significantly to morbidity and mortality. O-linked β-N-acetylglucosamine (O-GlcNAc) is a dynamic posttranslational modification that plays an important role in numerous biological processes, both in normal cell functions and disease. O-GlcNAc increases in response to stress. This increase mediates stress tolerance and cell survival, and is protective. Increasing O-GlcNAc is protective against IR injury. Experimental cellular and animal models, and also human studies, have demonstrated that protection against IR injury by ischemic preconditioning, and the more clinically applicable remote ischemic preconditioning, is associated with increases in O-GlcNAc levels. In this review we discuss how the principal mechanisms underlying tissue protection against IR injury and the associated immediate elevation of O-GlcNAc may involve attenuation of calcium overload, attenuation of mitochondrial permeability transition pore opening, reduction of endoplasmic reticulum stress, modification of inflammatory and heat shock responses, and interference with established cardioprotective pathways. O-GlcNAcylation seems to be an inherent adaptive cytoprotective response to IR injury that is activated by mechanical conditioning strategies.

## 1. Introduction

Stressors triggering organ damage and disease continuously influence the cells of biological organisms. Damage introduced by ischemia reperfusion injury (IR injury) is pivotal in diseases such as coronary heart disease (CHD) and stroke, causing substantial mortality and morbidity [1,2]. Restoration of blood flow to the ischemic area is the most important component for reduction of infarct size. Implementation of reperfusion modalities, including percutaneous coronary intervention and thrombolytic therapy, has significantly improved mortality and morbidity over the last 20 years [3,4]. However, CHD remains the leading cause of death in the Western world [5]. While reperfusion is essential for cytoprotection, it also contributes to cellular damage by inducing reperfusion injury [6]. The body has innate cytoprotective systems that activate protection against IR injury. Ischemic preconditioning (IPC) is a potential treatment modality that activates intrinsic protective mechanisms against IR injury. By short sublethal periods of ischemia and reperfusion, IPC activates a variety of protective mechanisms that render tissue or organs resistant to infarction from subsequent sustained ischemia and reperfusion [7]. This mechanism not only offers protection of the tissue subjected to brief ischemia, but also renders myocardium resistant to ischemia by exposure of a remote organ to an IPC stimulus [8,9]. This discovery has greatly facilitated the ability to translate this concept into a clinical applicability. Remote ischemic conditioning (RIC) by subjecting an arm to intermittent short periods of ischemia and reperfusion increases myocardial salvage in patients with evolving myocardial infarction [10], reduces release of troponin in patients undergoing heart surgery [11,12], and reduces cerebral damage in stroke patients [13].

One of the earliest responses to cellular stress is a rapid and global increase in the rate of O-GlcNAcylation, a dynamic posttranslational glycosylation of a variety of proteins [14]. This increase facilitates stress tolerance and cell survival, and has been shown to be protective [15]. An increase of intracellular O-GlcNAc levels protects against IR injury. Pharmacological and genetic augmentations of O-GlcNAc levels in isolated perfused hearts reduce infarct size when subjected to IR injury [16,17,18,19]. O-GlcNAc is also involved in protection against IR injury by ischemic conditioning. IPC has been shown to increase myocardial O-GlcNAc levels in rodents [20,21,22]. RIC has been shown to increase myocardial O-GlcNAc levels in human atrial trabeculae [23]. Blocking O-GlcNAcylation abrogated the cardioprotective effect of RIC in human atrial trabeculae [23].

The aim of the present review is to evaluate the impact of current research on the underlying mechanisms behind the interaction between O-GlcNAcylation and protection against IR injury. However, specific research on the effects of O-GlcNAcylation in IR injury is limited, so we critically evaluated whether the protective mechanisms associated with O-GlcNAcylation from studies conducted in other models may be extrapolated to the settings of IR injury.

## 2. O-Linked β-N-Acetylglucosamine (O-GlcNAc)

**Figure 1:**
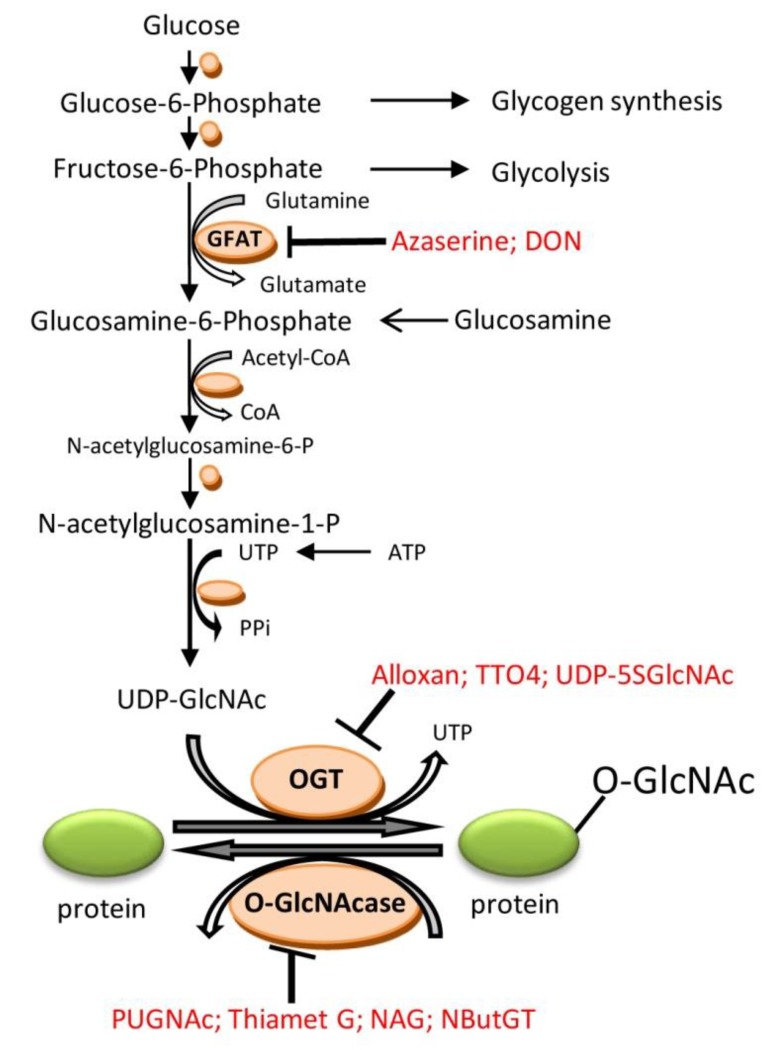
The hexosamine biosynthsis pathway (HBP) and protein O-GlcNAcylation. Enzymes are illustrated by orange circles. Blockers are written in red. Abbreviations: GFAT: l-glutamine-d-fructose 6-phosphate amidotransferase; UDP-GlcNAc: UDP-N-acetylglucosamine; OGT: uridine-diphospho-N-acetylglucosamine:polypeptide β-N-acetylglucosaminyltransferase; O-GlcNAcase: β-N-acetylglucosaminidase; DON: 6-diazo-5-oxo-norleucine; UDP-5SGlcNAc: uridine diphospho-5-thio-N-acetylglucosamine; TTO4: 2[(4-chlorophenyl)imino]tetrahydro-4-oxo-3-[2-tricyclo(3.3.1.13.7)dec-1-ylethel]; PUGNAc: O-(2-acetamido-2-deoxy-d-glucopyranosylidene)amino-N-phenylcarbamate; NAG:1,2-Dideoxy-2′-methyl-α-d-glucopyranoso-[2,1-d]-δ2′-thiazoline; NButGT:1,2-dideoxy-2′-propyl-α-d-glucopyranoso-[2,1-d]-δ2′-thiazoline. GFAT can be inhibited by glutamine analogue azaserine (O-diazoacetyl-L-serine) or DON. OGT can be inhibited by the uridine analogue alloxan, substrate analog of O-GlcNAc UDP-5SGlcNAc, or with TTO4, whereas O-GlcNAcylation of proteins can be rapidly increased by inhibiting O-GlcNAcase with PUGNAc, Thiamet G, NAG, or NButGT. Alloxan has also shown to have an inhibitory effect on O-GlcNAcase.

O-linked β-N-acetylglucosamine (O-GlcNAc) is a dynamic posttranslational modification of nuclear, cytoplasmatic, and mitochondrial proteins through the hexosamine biosynthetic pathway (HBP) (Figure 1).

The initial step of the HBP transforms fructose-6-phosphate to glucosamine-6-phosphate by the enzyme l-glutamine-d-fructose-6-phosphate amidotransferase (GFAT). Through several enzymatic steps, glucosamine-6-phosphate is converted into uridine-diphosphate-N-acetylglucosamine (UDP-GlcNAc), which acts as substrate for the enzyme that catalyzes the addition of O-GlcNAc to serine and threonine residues, uridine-diphospho-N-acetylglucosamine:polypeptide-N-acetylglucosaminyltransferase (OGT). The removal of O-GlcNAc is catalyzed by β-hexoamininidase (O-GlcNAcase). In experimental settings, the formation of O-GlcNAc can be augmented pharmacologically by increasing flux through HBP using glucosamine [24,25] or glutamine treatment [18], or pharmacologic inhibition of O-GlcNAcase with O-(2-acetamido-2-deoxy-d-glucopyranosylidene)amino-N-phenylcarbamate (PUGNAc) [24,26], Thiamet G [27], or 1,2-Dideoxy-2′-methyl-α-d-glucopyranoso[2,1-d]- δ2′-thiazoline (NAG-thiazoline) [16], or 1,2-dideoxy-2′-propyl-α-d-glucopyranoso-[2,1-d]-δ2′-thiazoline (NButGT) [25], and through genetic inhibition of O-GlcNAcase by transfection with short interfering (si) RNA directed against O-GlcNAcase [26] or genetic increase in OGT expression [28,29], by adenoviral overexpression. O-GlcNAcylation can be blocked by pharmacologic GFAT inhibition with the glutamine analogue azaserine (o-diazoacetyl-L-serine) [18,23,24], or GFAT inhibitor 6-diazo-5-oxo-norleucine (DON) [30], or OGT inhibition with uridine analogue alloxan [24,31] or with 2[(4-chlorophenyl)imino]tetrahydro-4-oxo-3-[2-tricyclo(3.3.1.13.7)dec-1-ylethel] (TTO4) [27], or substrate analog of O-GlcNAc uridine diphospho-5-thio-N-acetylglucosamine (UDP-5SGlcNAc) [32]. Unfortunately none of the enzyme blockers are entirely specific [22,33]. Alloxan has inhibitory effects on both OGT and O-GlcNAcase [33]. O-GlcNAcylation can also be blocked genetically by adenoviral overexpression of O-GlcNAcase [26], inhibition of OGT by transfection with short interfering (si) RNA directed against OGT [28], or genetic deletion of OGT using a cre-lox approach [28].

O-GlcNAc modification plays an important role in numerous biological processes, both in normal cell functions such as regulating cell cycle [34], protease activity [35], and transcription [36,37], and in the etiology of chronic disease. Transient elevation of O-GlcNAc in relation to stress is protective, as discussed below, while chronic elevation of O-GlcNAc plays a role in the pathophysiological processes of neurodegeneration in Alzheimer’s [38] and Parkinson’s disease[39]; cancer [40]; hypertension; cardiac hypertrophy and failure [41]; complications of diabetes mellitus, particularly insulin resistance [42]; increased atherosclerosis [43]; and cardiac dysfunction [44]. 

In mammalian cells multiple stress varieties, such as chemical, thermal, and biological stimuli, increase O-GlcNAcylation of nuclear and cytoplasmatic proteins. A reduction of O-GlcNAc levels, achieved by deletion of OGT, sensitizes cells to thermal stress, while augmentation of O-GlcNAc by genetic overexpression of OGT or pharmacologic inhibition of O-GlcNAcase with PUGNAc increases thermal tolerance and improves cell survival [14]. Accordingly, augmentation of intracellular O-GlcNAc levels is protective against IR injury in various experimental models. In neonatal rat ventricular myocytes, an increase of protein O-GlcNAcylation by inhibition of O-GlcNAcase pharmacologically with PUGNAc [24,26] or NAG-thiazoline derivative [25], or genetically by RNA interference of O-GlcNAcase [26], improves survival following IR injury. Moreover, an increase of O-GlcNAc levels by glucosamine treatment or genetic augmentation of OGT expression by adenovirus transfection attenuates cardiac myocyte death following hypoxia–reoxygenation [25,28]. Reduction in O-GlcNAc levels by pharmacologic (alloxan or TT04) [24,28] or genetic OGT inhibition [28], or genetic overexpression of O-GlcNAcase [26], exacerbated myocyte death.

In isolated perfused hearts, increasing cardiac O-GlcNAc levels by perfusion with glucosamine or glutamine [17,18], subjection to O-GlcNAcase inhibitor PUGNAc [19], or NAG-thiazoline derivatives [16] reduced myocardial damage when subjected to IR injury. Additional support for the cardioprotective effect of O-GlcNAc was given in studies demonstrating that the increase in myocardial O-GlcNAc levels and the protective effect of glucosamine was blocked by inhibition of OGT with alloxan or inhibition of GFAT with azaserine [17,18].

An increase in cerebral O-GlcNAc levels by glucosamine treatment has been shown to be neuroprotective against IR injury. In a rat middle cerebral artery occlusion model, intraperitoneal glucosamine administration reduced cerebral infarct size and afforded reduction in motor impairment and neurological deficits [45].

**Figure 2:**
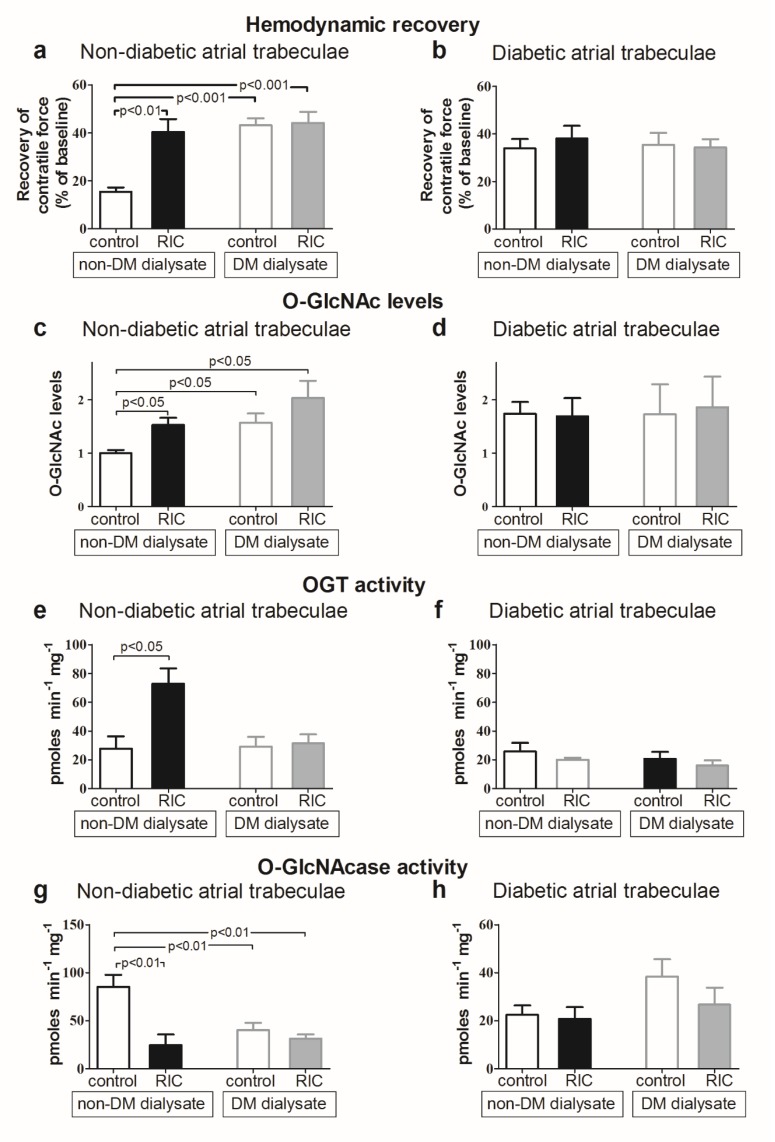
(**a**,**b**) Recovery of contractile force in atrial trabeculae from non-diabetic (**a**) and diabetic (**b**) patients, perfused with control and RIC dialysate from non-diabetic and diabetic patients; (**c,d**) O-GlcNAc levels in atrial trabeculae from non-diabetic (**c**) and diabetic (**d**) patients, perfused with control and RIC dialysate from non-diabetic and diabetic patients; (**e,f**) OGT activity in atrial trabeculae from non-diabetic (**e**) and diabetic (**f**) patients, perfused with control and RIC dialysate from non-diabetic and diabetic patients; (**g,h**) O-GlcNAcase activity in atrial trabeculae from non-diabetic (**g**) and diabetic (**h**) patients, perfused with control and RIC dialysate from non-diabetic and diabetic patients. Data are mean ± SEM. Originally published *Cardiovasc Res.* 2013 Feb 1; 97(2): 369–378 [23]. Reproduced with permission.

In 2008, Jones et al. used a mouse model to demonstrate that IPC increased cardiac O-GlcNAc levels and reduced infarct size after 30 min of left coronary artery ligation and 24 h of reperfusion [20]. In an isolated perfused rat heart model, we confirmed in two studies that IPC reduces infarct size and increases cardiac O-GlcNAc levels [21,22]. In the first study, the increase in O-GlcNAc was induced by enhanced OGT expression and activity [22]. In the second study, we demonstrated that cardioprotection by IPC was associated with increased myocardial glucose uptake, which may also contribute to the mechanism by which IPC increases O-GlcNAc levels [21]. To test the influence of O-GlcNAc in protection by the more clinically relevant RIC, we demonstrated that dialysate from healthy volunteers exposed to RIC improved post-ischemic recovery and increased myocardial O-GlcNAc levels in human isolated atrial trabeculae subjected to IR injury (Figure 2) [23]. The cardioprotective effect and the increase in O-GlcNAc were abolished by the GFAT inhibitor azaserine. In human non-diabetic trabeculae, the increase in O-GlcNAc afforded by dialysate from healthy volunteers subjected to RIC was associated with increased OGT activity and decreased O-GlcNAcase activity [23].

In conclusion, an increase of cardiac O-GlcNAc levels is protective against IR injury. The protection has been demonstrated in many different models, including cells, isolated heart models, and in vivo. Protection against IR injury following IPC and RIC is associated with increase in O-GlcNAc levels, predominately through increased OGT activity and increased glucose uptake.

## 3. Mechanisms by Which O-GlcNAc Confers Protection

### 3.1. Calcium Overload

Calcium overload contributes to the detrimental cascade of IR injury. Similar to the effect of IPC [46,47], increasing O-GlcNAc levels by glucosamine treatment protected against injury resulting from calcium paradox [17]. The calcium paradox was established in isolated perfused rat hearts, where calcium-free perfusion followed by perfusion with buffer containing physiological calcium concentration led to cardiomyocyte injury [48,49]. Glucosamine treatment also blocked ANG-II-induced calcium overload in neonatal rat ventricular myocytes [31]. The beneficial effects were dependent on OGT [31]. More importantly, O-GlcNAc also attenuates calcium overload in IR injury. In neonatal rat ventricular myocytes, glucosamine treatment and OGT overexpression increased O-GlcNAc levels and attenuated hypoxia-induced calcium overload during reoxygenation, when assessed by time-lapse fluorescence microscopy [24,50]. O-GlcNAcylation is known to be one of the regulators of the inositol 1,4,5-trisphosphate (InsP3) receptor type I, a channel for intracellular calcium release in many cell types [51].

**Figure 3:**
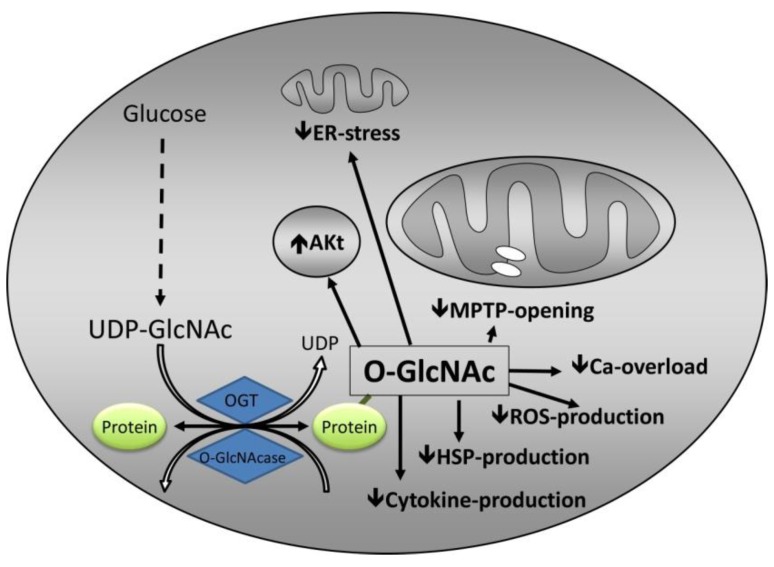
Summary of potential mechanisms by which O-GlcNAc confers protection. The mechanisms involve attenuation of endoplasmic reticulum (ER) stress, interaction with established cardioprotective pathways, predominantly Akt, inhibition of mitochondrial permeability transition pore (MPTP), attenuation of calcium overload, reactive oxygen species (ROS), heat shock protein (HSP), and cytokine production that reduce systemic inflammatory response. Other abbreviations as in Figure 1.

In conclusion, O-GlcNAc may be involved in protection against IR injury through attenuation of calcium overload (Figure 3). The mechanisms by which O-GlcNAc attenuates calcium overload are not known. O-GlcNAc may regulate other calcium channels in the endoplasmic reticulum [51,52] or mitochondria, but currently no evidence documents this speculation.

### 3.2. mPTP Opening

Opening of the mitochondrial permeability transition pore (mPTP) is considered to be a critical step in cellular death from IR injury. Opening of the mPTP causes depolarization of the mitochondria, influx of solutes and water, mitochondrial swelling, rupture, and release of pro-apoptotic factors as cytochome C [53,54,55].

The effect of O-GlcNAcylation on ROS generation in the setting of IR injury has been sparsely evaluated. It has been demonstrated that augmenting O-GlcNAc levels by adenoviral OGT overexpression or PUGNAc treatment attenuated hypoxia and oxidative stress-induced ROS generation [50]. Notably, in contrast to this study, O-GlcNAcylation is thought to promote ROS generation in models of hyperglycemia and glucose toxicity [56]. The interplay between O-GlcNAc and ROS is complex and not fully understood [57,58]. In the setting of chronic elevation of O-GlcNAc by hyperglycemia or glucosamine treatment, ROS generation was elevated and cell death induced [59], while more acute increase in O-GlcNAcylation attenuated ROS generation [50].

Increased intracellular O-GlcNAc levels attenuate the loss of mitochondrial membrane potential. In neonatal rat cardiac myocytes, augmentation of O-GlcNAc levels by treatment with PUGNAc, glucosamine, OGT overexpression, or O-GlcNAcase inhibition with a NAG-thiazoline derivative significantly attenuated loss of mitochondrial membrane potential in a dose-dependent manner after exposure to H_2_O_2_, as assessed by fluorescent cationic dye, JC-1, or TMRE fluorescence [20,25]. Increased O-GlcNAc levels also attenuated IR-induced loss of mitochondrial membrane potential. Genetic overexpression of OGT, or inhibition of O-GlcNAcase, increased O-GlcNAc and protected neonatal rat cardiac myocytes from cell death following hypoxia–reoxygenation, and aggravated the loss of mitochondrial membrane potential assessed by changes in TMRE fluorescence. Pharmacologic inhibition of OGT with TT04 exacerbated the loss of mitochondrial membrane potential and cell death [26,28].

Substantial evidence documents that increased cardiac O-GlcNAc levels attenuate mPTP opening [20,25,28,50,60]. Ngoh et al. demonstrated that in neonatal rat ventricular myocytes, increased cardiac O-GlcNAc levels by OGT overexpression significantly delayed H_2_O_2_ mediated mPTP opening, as assessed by changes in calcein fluorescence using time-lapse fluorescence microscopy, while O-GlcNAcase overexpression accelerated H_2_O_2_ mediated mPTP formation [50]. O-GlcNAcase inhibition with PUGNAc significantly mitigated mPTP formation [50]. Hirose et al. showed that isoflurane treatment was cardioprotective in mice and in cardiomyocytes, and was associated with elevation in O-GlcNAc levels [60]. The cardioprotective effect and increase in O-GlcNAc levels were eliminated by the OGT inhibitor alloxan. Similar to Ngoh’s findings, Hirose showed that the cardioprotective effect was associated with a delay of H_2_O_2_ mediated mPTP opening in isolated adult cardiomyocytes, and the effect was abrogated by OGT inhibition [60]. Following IR of isolated neonatal rat ventricular myocytes, increasing O-GlcNAc levels by glucosamine treatment, OGT overexpression, and O-GlcNAcase inhibition using a NAG-thiazoline derivative decreased cytochrome C release, reflecting attenuated mPTP opening [25]. Increasing cardiac O-GlcNAc levels enhanced the resistance of isolated mitochondria cardiomyocytes towards calcium-induced mitochondrial swelling [20], also reflecting attenuated mPTP opening. Accordingly, reduction in cardiac O-GlcNAc levels sensitized isolated mitochondria to calcium-induced mitochondrial swelling [28].

The mechanism by which O-GlcNAc inhibits mPTP opening is not known, mainly because the exact structure of the mPTP is not established and the identity and function of a variety of subunits are not clear. The mPTP is thought to consist of several subunits that include several factors modifying the transporter function. It is widely accepted that the pore consists of the voltage-dependent anion channel (VDAC) in the mitochondrial outer membrane, the adenine nucleotide translocase (ANT) in the mitochondrial inner membrane, the matrix protein cyclophilin D, and complex V ATP synthase. Interaction between these subunits increases the sensitivity of the mPTP to calcium. The interaction can be prevented in several ways [61]. Cyclosporine A inhibits mPTP opening through cyclophilin D inhibition [62], while anti-apoptotic factors such as Bcl-2 interact with ANT to hinder opening of the mPTP [61].

Mitochondrial subunit VDAC is subject to O-GlcNAc modification. In isolated cardiomyocytes, PUGNAc elevated global O-GlcNAc levels and increased O-GlcNAcylation of the VDAC subunit, while the cardiomyocytes simultaneously became more resistant to calcium-induced swelling compared with those treated with vehicle [20,28,60]. O-GlcNAc also influences Bcl-2 levels. In neonatal rat ventricular myocytes, glucosamine and OGT transfection increased O-GlcNAc levels, attenuated cell injury following IR injury, increased mitochondrial Bcl-2 levels, and attenuated mitochondrial-mediated apoptosis evaluated by assessment of cytochrome C loss [25]. Whether O-GlcNAcylation of VDAC or the O-GlcNAc induced increase in Bcl-2 prevents a direct interaction between the subunits and the mPTP opening is not known. 

Taken together, available data indicate that increased O-GlcNAc protects against IR injury by attenuation of mPTP opening (Figure 3). The mechanisms are not fully understood, and it is not known whether a direct effect of increased intracellular O-GlcNAc content and O-GlcNAcylation of mPTP subunits, or an indirect effect through attenuation of calcium overload and ROS production prevails.

### 3.3. Endoplasmic Reticulum Stress

The endoplasmic reticulum (ER) maintains synthesis, folding, and transport of proteins. Exposure to stress, such as IR, provokes ER stress, potentially leading to increases in unfolded proteins and subsequent apoptosis [63,64]. Activation of the unfolded protein response (UPR) promotes re-establishment of normal ER function, but prolonged ER stress activates a maladaptive response of UPR ultimately leading to apoptosis. The transcription factor CCAAT-enhancer-binding protein homologous protein (CHOP) is also involved in ER stress-induced apoptosis [65,66], and PERK-mediated phosphorylation of eIF2α is thought to play a dominant role in the induction of CHOP in response to ER stress [67,68].

O-GlcNAc mediated protection against IR injury may partially be mediated by modulation of ER stress. In neonatal rat ventricular myocytes, pharmacological O-GlcNAcase inhibition using PUGNAc augmented O-GlcNAc levels and reduced brefeldin A-induced ER stress, according to UPR-inducible proteins Grp94, Grp78, and calreticulin levels. Increased O-GlcNAc levels blocked the activation of maladaptive ER stress response as reflected by diminished CHOP activation, and mitigated ER stress-induced cell death but not apoptosis, according to caspase-3/7 activity [29]. In a rabbit model of renal ischemia, glucosamine treatment increased O-GlcNAc levels and protected against renal damage. Glucosamine attenuated increase in CHOP and GRP78 expression during hypoxia, and decreased the proportion of apoptotic cells [69].

O-GlcNAc may protect cells against ER stress-induced apoptosis through regulation of eIF2α phosphorylation. In HepG2 cells, augmentation of O-GlcNAc levels by Thiamet G treatment or OGT overexpression showed that eIF2α was O-GlcNAcylated at Ser 219, Thr 239, and Thr 241, hindering phosphorylation of eIF2α and reducing CHOP activation [70]. Point mutation of the O-GlcNAcylation sites of eIF2α increased eIF2α phosphorylation and CHOP activation, and was associated with increased apoptosis upon ER stress [70].

Overall, O-GlcNAc mediated protection against IR injury seems to involve a reduction of ER stress (Figure 3) through attenuated induction of CHOP, presumably by inhibition of eIF2α phosphorylation.

### 3.4. Inflammation

IR injury induces an inflammatory response with release of cytokines, activation of the complement system, and activation of neutrophils [71]. IR also activates Nuclear Factor kappa-B (NF-κB) during reperfusion, which may have a detrimental effect, as inhibition of NF-κB improves outcome after IR injury [72,73]. In contrast, NF-κB activation may have a beneficial role in IPC. A preconditioning stimulus seems to activate NF-κB, and pharmacological inhibition of NF-κB abolishes the cardioprotective effect of preconditioning [74,75]. The finding is ambiguous because protection in some models, including models of non-cardiac IPC, is associated with depression of NF-κB [76,77,78].

O-GlcNAc has anti-inflammatory effects in a variety of cell types and models [79,80,81,82].

In a model of endoluminal arterial injury, the increase of proinflammatory mediator expression (chemokines and adhesion molecules) was attenuated, and glucosamine or PUGNAc reduced infiltration of leukocytes after balloon injury of the carotid artery [83].

Increased O-GlcNAcylation by glucosamine or Thiamet G administration has a neuroprotective effect against IR injury through suppression of inflammation. Glucosamine and Thiamet G treatment suppress induction of proinflammatory markers, IL1b, IL6, TNF-α, iNOS, and COX-2 [45,84]. Studies investigating the protection by O-GlcNAc through anti-inflammatory effects in classic IR injury models beyond neuroprotection are scarce.

In a trauma–hemorrhagic shock model, PUGNAc administration increased O-GlcNAc levels, improved cardiac function after resuscitation, and reduced plasma tumor necrosis factor-α (TNF-α) and interleukin-6 (IL-6) levels 2 h after resuscitation [85,86], while inflammatory cytokines did not differ 24 h after resuscitation [87]. In a similar model using not-resuscitated animals, glucosamine treatment had no effect on TNF-α and IL-10 release [88], suggesting that reperfusion is necessary for this protection mechanism to be involved. In a model of trauma–hemorrhagic shock and resuscitation, an increase of cardiac O-GlcNAc levels by glucosamine treatment was associated with attenuation of NF-κB activation. The nuclear translocation of NF-κB that leads to an inflammatory and immune response is dependent on the phosphorylation of IκB-α, which leads to degradation by the proteasome. Glucosamine attenuated the increase of IκB-α phosphorylation and the subsequent NF-κB nuclear translocation, as well as the attenuation of the increase of mRNA of TNF-α, IL-6, and ICAM-1 expression. Neutrophil infiltration was reduced, as evaluated by reduced MPO activity [89]. Similarly, in cultured cardiomyocytes, OGT overexpression attenuated LPS-induced increase in IκB-α phosphorylation, ICAM-1, TNF-α levels, and NF-κB activation, while increased O-GlcNAc levels by glucosamine treatment of mouse macrophage cells attenuated LPS-induced IκB-α phosphorylation and iNOS expression [89]. Potential mechanisms by which O-GlcNAc attenuates NF-kB activation are not clear, but may be by decreased IκB-α by inhibition of proteasome activity [90].

In vascular smooth muscle cells and human umbilical vein endothelial cells, increased O-GlcNAc levels by PUGNAc or glucosamine treatment attenuated the TNF-α induced activation of NF-κB [91,92]. Phosphorylation of the p65 subunit of NF-κB is required for the transcriptional activation of NF-κB. Glucosamine or PUGNAc treatment increased O-GlcNAcylation of p65 subunit of NF-κB, which attenuated the TNF-α-induced phosphorylation of p65 subunit of NF-κB, subsequently inhibiting activation of NF-κB and NF-κB mediated inflammatory response [91,92]. In a similar model, augmentation of O-GlcNAc protected against TNF-α induced oxidative stress and vascular dysfunction, while associated with inhibiting iNOS expression and suppressing the nitrotyros(yl)ation of proteins [93]. Additionally in neuroprotection, a potential mechanism by which O-GlcNAc protects against IR injury and O-GlcNAc regulates NF-κB activation could be by inhibiting translocation of p65 [84].

Thus, available data suggest that O-GlcNAc mediated protection against IR injury involves suppression of the inflammatory response through reduced induction of proinflammatory markers (Figure 3), and attenuated NF-κB activation by hindering IκB-α phosphorylation and translocation of p65.

### 3.5. Heat Shock Proteins

Heat shock proteins (HSP) are a group of proteins produced by the cell in response to stress stimuli. They play a crucial role as chaperones in cell-cycle control, stabilizing, repair, folding, and unfolding of proteins [84,94]. O-GlcNAc is involved in regulating the expression of numerous HSPs. Elevating O-GlcNAc levels augments expression of HSPs by heat stress [14,95], while decreasing O-GlcNAc suppresses expression of HSPs [96]. Expression of HSP70 and HSP72 may be protective against IR injury through repression of apoptosis [97,98,99], because they are upregulated by a RIC stimulus [100]. Jones et al. showed that HSP70 levels were increased in cardiomyocytes that were protected by PUGNAc against H_2_O_2_ injury [20]. Whether the augmentation of HSP70 was required for conferring protection was not determined. In a study by Wischmeyer et al., administration of glucosamine reduced infarct size after IR injury, but the authors found no change in HSP72 or HSP73 during the course of the experiment [101]. HSP70 physically interacts with O-GlcNAc proteins through a lectinic activity [102], such that O-GlcNAc seems to modify protein stability through specific interaction with 70-kDa-HSP members.

O-GlcNAc may induce cardioprotection by stabilizing protein structure. However, further studies are needed to determine the interaction between O-GlcNAc, HSP and protection against IR injury.

### 3.6. Interaction with Established Cardioprotective Pathways

The reperfusion injury salvage kinase pathway (RISK) conveys the cardioprotective stimulus by IPC from the cell surface to the mitochondria through activation of survival kinases and cytokines in the beginning of reperfusion. IPC activates PI3K, Akt, and downstream kinases such as GSKβ by phosphorylation in a biphasic manner after the IPC stimulus, and in the beginning of reperfusion [103,104]. Knowledge about the interaction between O-GlcNAc and PI3K/Akt signaling in relation to protection against IR injury is scarce. An interplay between O-GlcNAc and Akt phosphorylation has been acknowledged, but the biological significance has not been fully characterized and understood [105]. While O-GlcNAcylation has been reported in some studies to impair the Akt phosphorylation, resulting in insulin resistance [106] and induction of apoptosis [107,108], studies in other settings report that the interplay between O-GlcNAc and Akt inhibits apoptosis and provide cytoprotection in the kidney [109] and the liver [110].

Hu et al. suggested that acute augmentation of O-GlcNAc confers renal protection through activation of PI3K/Akt. In this study, administration of glucosamine increased O-GlcNAc levels and conferred renal protection against contrast-induced acute kidney injury. Augmented O-GlcNAc signaling increased phosphorylation of Akt in Ser473, but not in Thr 308 and Thr450, and GSK-3β in Ser9. The OGT inhibitor alloxan and the PI3K inhibitors Wortmannin and LY294002 blocked the renoprotective effect. Immunoprecipitation demonstrated that O-GlcNAc modified Akt activity in renal tissue [109]. In isolated cardiomyocytes subjected to hypoxia/reoxygenation stimulus, Akt phosphorylation was increased during the first 30 min of reoxygenation. Pretreatment with Thiamet G increased cell survival with no change in activation of Akt. Akt phosphorylation during reoxygenation was enhanced by O-GlcNAcase inhibition with TT04, while cell survival decreased it [27]. Hence, activation of Akt does not appear to be a mechanism of Thiamet G-mediated cytoprotection. However, Akt phosphorylation may not have been measured in a timely way during reoxygenation, compared to the changes in Akt described in the RISK pathway [101,103].

Other established pathways in protection against IR injury by IPC are the survivor activating factor enhancement (SAFE) pathway [111] and nitric oxide (NO) pathway. O-GlcNAcylation generally suppresses induction of TNF-α, which is activated in IPC [112]. The interplay between O-GlcNAc and signal transducer and activator of transcription or NO-pathways has not to our knowledge been investigated in the IR injury.

Thus, cardioprotection involves an interaction between O-GlcNAc and Akt (Figure 3), but it is not clear whether O-GlcNAc mediated protection involves the RISK pathway. 

### 3.7. Impact of O-GlcNAcylation on Cardiac Function

As in skeletal muscle (for recent review see [113]), several contractile and regulatory proteins of cardiac muscle have been identified as being O-GlcNAcylated [114]. These observations strongly support the idea that O-GlcNAcylation is involved in the regulation of contraction, or in its dysfunction. O-GlcNAc is essential for cell viability. While acute activation of pathways that increase O-GlcNAc levels improves tolerance of cells to a wide range of stress stimuli, sustained increase in O-GlcNAc levels has been implicated in chronic disease states, in particular as a pathogenic contributor to insulin resistance [42] and diabetic complications [115]. The consequences of chronically elevated O-GlcNAc linked to metabolic disease may compromise cardiomyocyte [44] as well as vascular function [116]. The contrasting beneficial effect of acute elevation of O-GlcNAc levels demonstrate that this posttranslational modification system is extremely dynamic. However, the fundamental mechanisms involved in regulating O-GlcNAc turnover and the precise regulation of its functional consequences still remain limited.

### 3.8. Impact of Diabetes Mellitus on O-GlcNAcylation and Cardioprotective Efficacy

Diabetes mellitus is associated with increased O-GlcNAcylation of intracellular proteins in several cells and tissues, including cardiomyocytes [23], renal cells [117], pancreatic cells [118], erythrocytes [119], and cornea [120]. Chronically increased O-GlcNAcylation contributes to complications of diabetes mellitus, particularly insulin resistance [42], increased atherosclerosis [43], and cardiac dysfunction [44].

Studies in animal models have demonstrated that diabetic animals have reduced susceptibility to IR injury [121,122,123,124,125,126]. The tolerance is dependent on diabetes duration and the severity of diabetes [122,126]. We documented that O-GlcNAc levels were elevated in atrial trabeculae from diabetic patients, which was associated with improved hemodynamic function of the trabeculae. Perfusion of atrial trabeculae from non-diabetic patients with dialysate from diabetic volunteers increased O-GlcNAc levels, also conferring protection against IR injury (Figure 2) [23]. No further increase in O-GlcNAc levels and no additional protection were achieved by RIC in trabeculae perfused with dialysate from diabetic patients, or in trabeculae from diabetic patients, suggesting that type 2 diabetes per se activates, through augmentation of O-GlcNAc levels, an inherent cardioprotective mechanism that may restrict further cardioprotection by RIC [23]. In a study of isolated hearts from young Zucker diabetic fatty rats, we found a upwards tendency, although not a statistically significant increase, in O-GlcNAc levels compared with non-diabetic rats. Diabetic rats were endogenously protected with significant reduced infarct size after IR injury during normoglycemic conditions, but not during hypoglycemia [21]. The association between IPC, infarct size reduction, and increase of O-GlcNAc levels was found in both diabetic and non-diabetic animals [21]. In streptozocin-induced diabetic isolated perfused hearts, diabetes improved recovery after exposure to the calcium paradox. The improvement was associated with an increase in cardiac O-GlcNAc levels [17].

Altogether, the chronic elevation of O-GlcNAcylation in diabetes mellitus is associated with complications of diabetes, but also seems to be involved in endogenous protection against IR injury.

### 3.9. Pharmacological Modulation of O-GlcNAcylation and Role for Cardioprotection

The most clinically applicably pharmacological modulation of O-GlcNAc levels can be achieved by glutamine or glucosamine treatment, because both compounds increase O-GlcNAcylation and induce cardioprotection against IR injury in rodents in vivo and in isolated heart models [18,19,24,25,101], and rodents exposed to trauma hemorrhage [85,87,88,89]. In contrast, glutamine failed to provide any protective effect in pigs [127]. Cardiac O-GlcNAc levels were not measured in this study, so it cannot be ruled out that the lack of protection was due to an insufficient dose. The effect of oral glucosamine treatment remains controversial [128], which may be due to insufficient intestinal absorption to secure the needed increase in cardiac O-GlcNAc levels to induce cytoprotection.

Metformin induces cardioprotection against IR injury in the rat heart 24 h after administration [129]. The cardioprotective effect was associated with an increased AMPK activity, which is thought to be an important signal mediator of the cardioprotective effects by metformin. Although speculations about modification of O-GlcNAcylation may be attractive because metformin increases cellular glucose uptake, no available data support this assumption. In a streptozotocin-induced diabetic mouse model, eight days of metformin administration protected against retinal cell death by OGT inhibition and the interaction between OGT and NF-κB [130]. While NF-κB is an important regulator of programmed cell death, and elevated O-GlcNAc levels enhance NF-κB signaling, the findings may have implications for chronic glucotoxicity, but they can hardly be extrapolated to acute IR injury. While studies of glucose-insulin-potassium (GIK) infusion to patients undergoing cardiac surgery have shown a cardioprotective effect [131,132], GIK infusion to patients with ST-elevation myocardial infarction (STEMI) has yielded conflicting results about cardioprotective efficacy [133,134,135]. High dose GIK therapy offers no clinical benefit at 1 year in patients with STEMI without signs of heart failure treated with reperfusion therapy [136]. GIK treatment was associated with a significant increase in O-GlcNAcylation of selected protein bands in the Hypertrophy, Insulin, Glucose and Electrolytes (HINGE) trial [137]. While the underlying mechanisms may be several, including improved myocardial energy production efficiency during acute ischemia by high-dose glucose substituting depleted myocardial potassium levels during ischemia and suppression of circulating levels and myocardial uptake of free fatty acids, which are toxic to ischemic myocardium by insulin, the increased O-GlcNAcylation may be an additional mechanism that may explain the beneficial effects of GIK solution. Indeed, a recent experimental study demonstrated that glucose and insulin synergistically reduced ROS production, protected neonatal rat ventricular myocytes dose-dependently from apoptosis, and altered O-GlcNAc and OGT expression [138]. Efficacy seems to be dependent on early administration before reperfusion, and seems to be associated with activation of cardioprotective pathways rather than modulation of metabolism [139,140].

Volatile anesthetics have protective effects against IR injury [141,142,143]. The cardioprotective effect of isoflurane is associated with increase in cardiac O-GlcNAc levels and abrogated by OGT inhibitor alloxan [60]. Isoflurane induces O-GlcNAc modification of mitochondrial voltage-dependent anion channel. This modification inhibits the opening of the mPTP, and confers resistance to ischemia-reperfusion stress.

In conclusion, available data do not identify specific pharmacological compounds that convincingly establish them as cardioprotective drugs by modulation of the HBP activity at present. Increase of cardiac O-GlcNAc may be involved in the mechanism underlying cardioprotection by volatile anesthetics, and supports the idea that pharmacologic modulation of O-GlcNAcylation may be a potential target for future pharmacologic treatment.

## 4. Conclusions

Increased intracellular O-GlcNAc content is cytoprotective against a variety of stress stimuli, including protection against IR injury. Studies on the involvement of O-GlcNAc in IPC are limited, but cardioprotection against IR injury by IPC seems to be associated with increased O-GlcNAc levels. Several of the mechanisms through which O-GlcNAc affords protection correlate with mechanisms underlying IPC, including attenuation of calcium overload, inhibition of mPTP opening, reduction of ER stress and apoptosis, suppression of the inflammatory response, and HSP expression. Thus, O-GlcNAcylation seems to be an inherent adaptive cytoprotective response to IR injury that may be activated by mechanical conditioning strategies.

## Abbreviations

ANT	Adenine nucleotide translocase
CHD	Coronary heart disease
CHOP	CCAAT-enhancer-binding protein homologous protein
DON	6-diazo-5-oxo-norleucine
ER	Endoplasmic reticulum
GFAT	l-glutamine-d-fructose-6-phosphate amidotransferase
GIK	Glucose-insulin-potassium
HBP	Hexosamine biosynthetic pathway
HSP	Heat shock proteins
IL-6	Interleurkin-6
IPC	Ischemic preconditioning
IR	Ischemia-reperfusion
mPTP	Mitochondrial permeability transition pore
NAG	1,2-dideoxy-2′-methyl-α-d-glucopyranoso-[2,1-d]-δ2′-thiazoline
NButGT	1,2-dideoxy-2′-propyl-α-d-glucopyranoso-[2,1-d]-δ2′-thiazoline
NF-κB	Nuclear factor kappa-B
NO	Nitric oxide
O-GlcNAc	O-linked β-N-acetylglucosamine
OGT	Uridine-diphospho-N-acetylglucosamine:polypetptide-N-acetylglycosaminyltransferase
O-GlcNAcase	β-N-hexoamininidase
PUGNAc	O-(2-acetamido-2-deoxy-d-glucopyranosylidene)amino-N-phenylcarbamate
RISK	Reperfusion Injury Salvage Kinase pathway
RIC	Remote ischemic conditioining
SAFE	Survival activating factor enhancement
STEMI	ST-elevation myocardial infarction
TNF-α	Tumor necrosis factor-α
TTO4	2[(4-chlorophenyl)imino]tetrahydro-4-oxo-3-[2-tricyclo(3.3.1.13.7)dec-1-ylethel]
UDP-5SGlcNAc	Uridine diphospho-5-thio-N-acetylglucosamine
UDP-GlcNAc	Uridine-diphosphate-N-acetylglycosamine
VDAC	Voltage-dependent anion channel
